# Influence of Uniaxial Stress on the Shear-Wave Spectrum Propagating in Steel Members

**DOI:** 10.3390/s19030492

**Published:** 2019-01-25

**Authors:** Zuohua Li, Jingbo He, Diankun Liu, Nanxi Liu, Zhili Long, Jun Teng

**Affiliations:** Harbin Institute of Technology Shenzhen, Shenzhen 518055, China; lizuohua@hit.edu.cn (Z.L.); hejingbo@stu.hit.edu.cn (J.H.); liudiankun2010@163.com (D.L.); liu_nanxi@foxmail.com (N.L.); longzhili@hit.edu.cn (Z.L.)

**Keywords:** uniaxial stress measurement, structural steel members, amplitude spectrum, phase spectrum, shear-wave birefringence, acoustoelastic effect

## Abstract

Structural health monitoring technologies have provided extensive methods to sense the stress of steel structures. However, monitored stress is a relative value rather than an absolute value in the structure’s current state. Among all the stress measurement methods, ultrasonic methods have shown great promise. The shear-wave amplitude spectrum and phase spectrum contain stress information along the propagation path. In this study, the influence of uniaxial stress on the amplitude and phase spectra of a shear wave propagating in steel members was investigated. Furthermore, the shear-wave amplitude spectrum and phase spectrum were compared in terms of characteristic frequency (CF) collection, parametric calibration, and absolute stress measurement principles. Specifically, the theoretical expressions of the shear-wave amplitude and phase spectra were derived. Three steel members were used to investigate the effect of the uniaxial stress on the shear-wave amplitude and phase spectra. CFs were extracted and used to calibrate the parameters in the stress measurement formula. A linear relationship was established between the inverse of the CF and its corresponding stress value. The test results show that both the shear-wave amplitude and phase spectra can be used to evaluate uniaxial stress in structural steel members.

## 1. Introduction

### 1.1. Absolute Stress in Structural Steel Members

Many large-scale steel structures have been built worldwide due to their high degree of industrialization [1,2]. Fully understanding the performance degradation of steel structures during their entire life cycle has become a significant topic [3], which has received increasing attention in academic and engineering fields [4,5]. Structural health monitoring [6,7] is one of the most effective technologies to sense the real response of the monitored objects. Many excellent monitoring technologies [8,9], systems [10], and advanced intelligent algorithms [11] have been developed and applied to solve engineering problems. A stress monitoring system [12,13], which plays an important role in structural health monitoring technologies, has been regarded as a mature way to obtain structural stress information from the macroscale stress distribution of a whole structure [14] to the microscale stress concentration of a local member [15]. However, the monitored stress value using a stress monitoring system is a relative value rather than an absolute value. The absolute stress, which represents the current state of structures, is a significant indicator for judging the safety of structures [16].

Existing stress measurement methods, such as diffraction [17,18] and magnetic methods [19], can be used to detect the absolute stress of materials. However, these methods are unable to adequately test large-scale steel members and are unsuitable for field applications because a strict testing environment is required during the testing process. In addition, the testing equipment is complex, and the testing process is time consuming. Generally, absolute stress measurements of structural steel members using structural health monitoring technologies remains a challenging task [20,21].

### 1.2. Ultrasonic Stress Measurement Methods

In recent years, ultrasonic methods, which are based on acoustoelastic effects, have been studied to evaluate the internal and initial stress in complex structures [22,23,24,25]. Compared with other stress measurement methods, such as X-ray diffraction [17], neutron diffraction [18], and magnetic [19] methods, ultrasonic methods have shown great prospects for use in in-site stress measurements [26]. Essentially, ultrasonic methods establish a linear relationship between the stress and ultrasonic wave velocities, that is, a time-of-flight (TOF) measurement [27]. Compared with other ultrasonic waves, a longitudinal critically refracted (Lcr) wave exhibits the greatest sensitivity to stress [28]. Hence, these waves have been widely used to evaluate welding residual stress [29], rail stress [30], steam turbine disk stress [31], and steel member stress [22]. To improve the signal-to-noise ratio, the laser-generated Lcr wave method was presented to evaluate the stress in a noncontact manner [32], and the piezoelectric effect-generated Lcr wave was investigated to detect the stress in an immersion manner [33]. Combining the experimental and the numerical analysis results, the colored stress distribution nephogram of a tested member can be sketched [34]. Because the Lcr wave energy is relatively small and rapidly decays, guided ultrasonic wave methods have been proposed and used to monitor the stress in steel strands [35] and aluminum plates [36,37], which is a further application of the acoustoelastic effect. Recently, the influence of a uniaxial load on the electromechanical impedance of embedded piezoceramic transducers in steel fiber concrete was investigated [38]. A normalized root-mean-square deviation index was developed to analyze the electromechanical impedance information, and the experimental results showed that the index increases with the uniaxial load, thus providing a potential method to evaluate the uniaxial stress of steel fiber concrete.

In addition to the methods described above, the shear wave [39] can also be used to evaluate stress. The effect of birefringence [40] describes a phenomenon in which the velocity of a shear wave varies when the shear wave vibrates in different directions, which endows the shear wave with unique advantages to evaluate the stress in materials. If two individual stress values in plate-like components need to be detected, then the combination of longitudinal and polarized shear waves is advantageous [41]. In addition, by measuring the velocities of the shear wave in two different polarization directions, the influence of texture during the stress evaluation can be separated [42]. Note that the aforementioned shear-wave methods are based on TOF measurements. The accuracy of the ultrasonic stress evaluation results is influenced by the TOF data collection. In fact, many uncertain factors, such as microcracks [43], inhomogeneous materials [44], coupling conditions [45], and temperature [46], may lead to a distortion of the waveform, which limits the industrial application of ultrasonic methods. It is critical to distinguish the influence of the uncertainty factors from that of stress [47]. To date, only a few systems have been used in practical engineering [48].

In addition to the above methods, shear-wave frequency domain signals have received attention in recent years. Shear-wave spectrum analysis methods are based on acoustoelastic theory and the shear-wave birefringent effect. When a beam of a shear wave is perpendicularly incident to a stressed solid, it separates into two modes. The two separated shear-wave modes travel with different velocities, which produces interference effects. The received shear-wave spectrum contains the interference information, which can be used to evaluate the absolute stress in solids [49]. Recently, the shear-wave amplitude spectrum method was proposed to measure the absolute stress in steel members [24]. The experimental results showed that the inverse of the CF linearly changed with the applied uniaxial stress, and then the mechanically applied stresses of the structural steel members were evaluated. The ultrasonic shear-wave amplitude spectrum method makes use of the amplitude spectrum to establish the relationship between the stress and the CF. In fact, the phase spectrum also contains the stress information along the shear-wave propagation path, which may provide a new method to detect the absolute stress in structural steel members. However, the effect of stress on the phase spectrum is not as well understood, which is the focus of this study.

### 1.3. Goals and Objectives of This Study

In light of the challenges described above, here we investigate the influence of uniaxial stress on shear-wave spectrum propagation in steel members. Compared to our previous work [24], which aimed to measure the absolute stress using the shear-wave amplitude spectrum, this paper further studies the phase spectrum of a shear wave propagating in steel members. Moreover, the shear-wave amplitude spectrum and phase spectrum are compared in terms of CF collection, parametric calibration and absolute stress measurement principles, which represents an expansion of our previous method [24]. For this purpose, the theoretical formulas of the shear-wave pulse echo phase spectrum are derived. Accordingly, the relationship between the uniaxial stress and the CF is established. Three structural steel members are tested to investigate the effect of the applied uniaxial stress on the shear-wave amplitude and phase spectra. The parameters representing the quantitative relationship between the stress and the CF are calibrated using the experimental data. The results show that the amplitude and phase spectra have the potential to be used for stress monitoring of in-service structures.

## 2. Theory

### 2.1. Theoretical Derivation of the Shear-Wave Pulse Echo Spectrum

The theoretical expression of the shear-wave pulse echo spectrum is derived on the assumption that the steel member interface exerts no effect on the propagation of the shear wave. In addition, the steel member material is assumed to be isotropic and homogeneous as well as be elastic in its range. When a beam of ultrasonic shear-waves is perpendicularly incident on a steel member, the motion equation of the shear-wave propagating is

(1) $$u_{0}=y(t)=\sum_{i=0}^{n}A_{i}\cos(w_{i}t+j_{i}),(i=0,1,2,...,n),$$

where the shear-wave contains various components of the harmonic vibration, *u*_0_ and *A*_i_ are the amplitude of the vibration source and the amplitude of the *i*th component, respectively; *w_i_* and *φ_i_* are the angular frequency and initial phase of the *i*th component, respectively; and *t* is the vibration time.

Uniaxial stress in steel members can cause an acoustic anisotropy of the material; that is, stress causes the ultrasonic velocity to change when vibrating in different directions. When an ultrasonic shear-wave is perpendicularly incident on a steel member under a uniaxial stressed state, it separates into two shear-wave modes with one polarization direction parallel to the stress direction and the other mode perpendicular to the stress direction [49]. The motion equations of the two separated shear waves are [40]

(2) $$u_{1}(x_{3},t)=y(t-\frac{x_{3}}{v_{31}})\cos\theta ,$$

(3) $$u_{2}(x_{3},t)=y(t-\frac{x_{3}}{v_{32}})\sin\theta ,$$

where *x*_1_, *x*_2_ and *x*_3_ are axes of the Cartesian coordinate; the two separated shear waves propagate in the positive direction of *x*_3_; *v*_31_ and *v*_32_ are the velocities of the two shear-waves traveling in the direction of *x*_3_ with a particle vibration parallel to *x*_1_ and *x*_2_, respectively; and *θ* is the angle between the incident shear-wave direction and the *x*_1_ direction.

When the two separated shear waves travel from the starting point on one side of the steel member to the rear side, they will be reflected and travel back to the starting point. The synthesis of the two reflected shear waves is

(4) $$u_{r}(t)=y(t-\frac{x_{3}}{v_{3}{}_{1}})\cdot \cos^{2}\theta +y(t-\frac{x_{3}}{v_{3}{}_{2}})\cdot \sin^{2}\theta ,$$

where *y*(*t* − *x*_3_/*v*_31_) and *y*(*t* − *x*_3_/*v*_32_) contain the information of the two separated shear waves’ TOF delay and cos^2^*θ* and sin^2^*θ* contain the amplitude information of the two wave components that synthesize pulse echo in the incident direction. Let *M* = cos^2^*θ* and *N* = sin^2^*θ*; then Equation (4) can be simplified as the following form:

(5) $$u_{r}(t)=y(t-\frac{x_{3}}{v_{31}})\cdot M+y(t-\frac{x_{3}}{v_{32}})\cdot N.$$

*U*_0_ (*f*) and *U_r_* (*f*) are defined as the Fourier transforms of *y*(*t*) and *u_r_*(*t*), respectively. The synthesis of the two reflected shear waves in the frequency domain is [24]

(6) $$U_{r}(f)=U_{0}(f)\cdot L(\theta ,f),$$

(7) $$\begin{array}{ll} L(\theta ,f)= & \cos(2\pi f\frac{x_{3}}{v_{31}})\cdot M+\cos(2\pi f\frac{x_{3}}{v_{32}})\cdot N\\ & -i\left[\sin(2\pi f\frac{x_{3}}{v_{31}})\cdot M+\sin(2\pi f\frac{x_{3}}{v_{31}})\cdot N\right] \end{array}$$

where *L*(*θ*,*f*) is defined as the interference factor (IF). Equation (6) is the theoretical expression of the shear-wave pulse echo spectrum propagating in steel members. Note that Equation (6) is equivalent to Equation (4) and contains the interference information of the two separated shear waves.

### 2.2. Theoretical Derivation of the Shear-Wave Amplitude Spectrum

Equation (6) contains information on the amplitude spectrum and phase spectrum. By taking the modular operation on both sides of Equation (6), the theoretical formula of the shear-wave amplitude spectrum can be obtained, which is shown in the following formula.

(8) $$\left|U_{r}(f)\right|=\left|U_{0}(f)\right|\cdot \left|L(\theta ,f)\right|,$$

(9) $$\left|L(\theta ,f)\right|=\sqrt{1+2MN(\cos\left(2\pi Pf)-1\right)},$$

where |*L*(*θ*,*f*)| is the amplitude of the interference factor (AIF) with a value ranging from 0 to 1; *P* equals (*2l*/*v*_31_ − *2l*/*v*_32_), which is the TOF difference of the two separated shear waves.

The AIF is a periodic function of the frequency and polarized angle. When the AIF reaches a minimum, the frequency and the polarized angle can be solved.

(10) $$\left\{ \begin{array}{l} f^{*}=\frac{2N_{1}-1}{2P},(N_{1}=1,2,3,...)\\ \theta =\frac{N_{2}\pi }{4},\;(N_{2}=1,3,5,...) \end{array}\right..$$

In Equation (10), *f* * is defined as the CF. The CFs are defined as the first CF (*f*_1_*), the second CF (*f*_2_*), the third CF (*f*_3_*), …, when *N*_1_ equals 1, 2, 3, …, respectively.

Equation (8) shows that the amplitude spectrum is a product of the incident shear-wave amplitude spectrum and the AIF. The periodic values for the frequency and the polarized angle are 1/*P* and *π*/2, respectively. Particularly, when the shear-wave polarized angle is an odd multiple of *π*/4, the AIF reaches 0 at the minimum point. This finding indicates that the energy of the harmonic component with the frequency of (2*N*_1_ − 1)/2*P* decreases to 0. Correspondingly, the amplitude value in the amplitude spectrum with a frequency of (2*N*_1_ − 1)/2*P* decreases to 0. In Equation (9), *P* is the TOF difference of the two separated shear waves. Because the TOF difference of the two separated shear waves is determined by the uniaxial stress, the CF in Equation (10) is related to the uniaxial stress in the steel member. This effect establishes the foundation for detecting the uniaxial stress in steel members from the shear-wave amplitude spectrum.

### 2.3. Theoretical Derivation of the Shear-Wave Phase Spectrum

*φ*_0_(*f*) and *φ_r_*(*f*) are defined as the phase spectra of *U*_0_ (*f*) and *U_r_* (*f*), respectively. The spectra *U*_0_ (*f*) and *U_r_* (*f*) are complex functions. By combination with Equation (6), the following expression can be obtained.

(11) $$\varphi_{r}(f)=\varphi_{0}(f)+\varphi_{L}(f),$$

where *φ_L_*(*f*) is defined as the phase of the interference factor (PIF).

From Equation (7), the following formula can be obtained:

(12) $$\varphi_{L}(f)=-\pi f(\frac{2l}{v_{31}}+\frac{2l}{v_{32}})-\arctan((M-N)\cdot \tan(\pi fP)).$$

By substituting Equation (12) into Equation (11), the theoretical expression of the shear-wave pulse echo phase spectrum can be obtained.

(13) $$\varphi_{r}(f)=\varphi_{0}(f)-\pi f(\frac{2l}{v_{31}}+\frac{2l}{v_{32}})-\arctan((M-N)\cdot \tan(\pi fP)).$$

From Equation (13), the shear-wave pulse echo phase spectrum contains three parts. The first part, *φ*_0_(*f*), is the phase spectrum of the incident shear wave. The second part, −*πf*(*2l*/*v*_31_ + *2l*/*v*_32_), is the delayed phase values of the shear-wave propagating a length of 2*l*. The third part equals −arctan((*M* − *N*)·tan(*πfP*)), which is the phase value caused by the TOF difference for the two separated shear waves. When the amplitude of the separated shear waves is identical, that is, *N* equals *M*, the third part in Equation (13) is 0, and the corresponding shear wave polarized angle is 45°. In the following theoretical derivation, as the third part in Equation (13) is significant for CF collection, the shear wave polarized angle should not be 45°. Comparing Equations (8) and (11), the interference factor plays different roles in the amplitude and phase spectra. The AIF (|*L*(*θ*,*f*)|) and the PIF (*φ_r_*(*f*)) indicate the amplitude change and the phase change in the synthesis shear wave, respectively.

When the shear-wave pulse echo amplitude spectrum reaches a minimum value, the phase difference of the two separated shear-waves should be (2*N*_3_ − 1)*π*. Hence, the phase difference of the two separated shear-waves corresponds to the TOF difference, *P*, should be (2*N*_3_ − 1)*π*.

(14) $$2\pi fP=(2N_{3}-1)\pi ,\;(N_{3}=1,\;2,\;3,\;...).$$

The solution of Equation (14) is

(15) $$f^{*}=\frac{2N_{3}-1}{2P},\;(N_{2}=1,\;2,\;3,\;...),$$

which is identical to the CF derived from the amplitude spectrum.

Particularly, when the polarized angle of the shear-wave is 45°, the third part in Equation (13) is 0. Then, the shear-wave pulse echo phase spectrum is

(16) $$\varphi_{45^{\circ }}(f)=\varphi_{0}(f)-\pi f(\frac{2l}{v_{31}}+\frac{2l}{v_{32}}).$$

**Figure 1:**
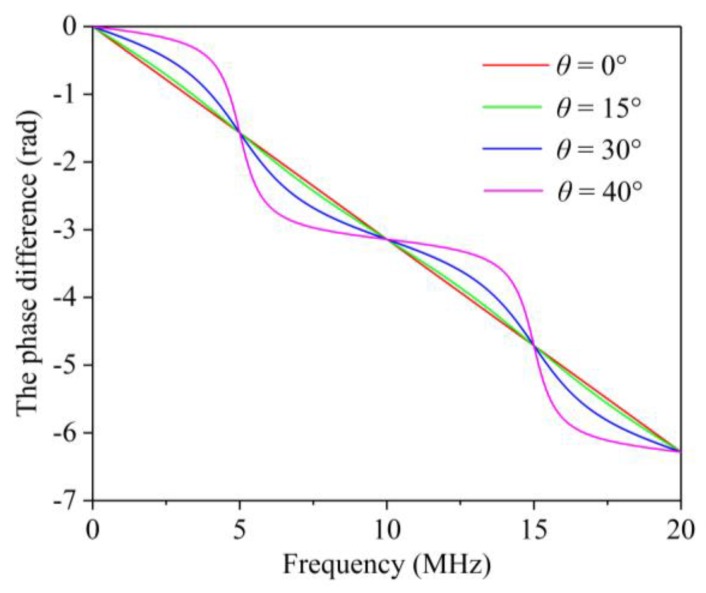
Functional image of the phase difference.

The phase difference between an arbitrary polarized angle *θ*, and the polarized angle of 45° is

(17) $$\Delta \varphi_{r}=-\arctan((M-N)\cdot \tan(\pi fP)).$$

where Δ*φ_r_* is defined as the phase difference (PD). A typical illustration of the PD is shown in Figure 1, in which *P* is taken as equal to 100 ns as an example. The CF in the curve of the PD corresponds to an inflection point. The inflection point in the PD curve can be used to identify the CF.

**Figure 2:**
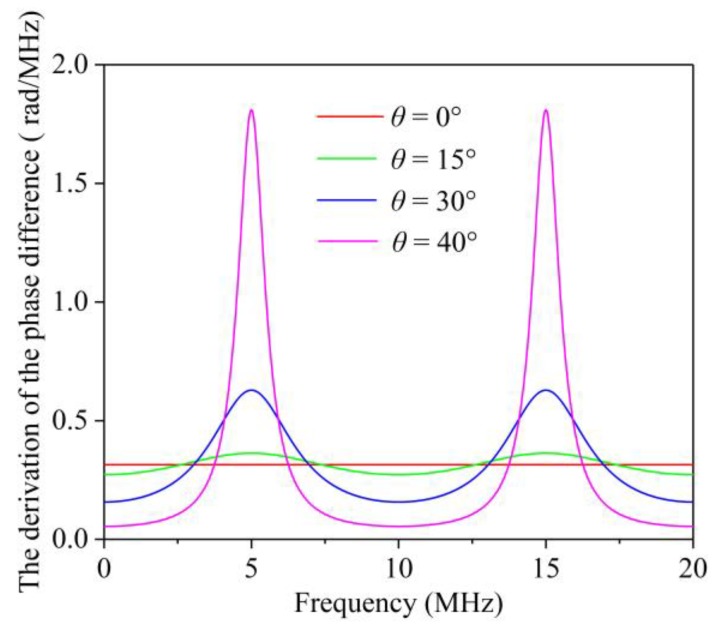
An illustration of the derivation of the phase difference.

A method for obtaining the inflection point is to draw an image of the derivation of the PD (DPD), in which the maximum point corresponds to the CF. An illustration of the DPD curve when *P* = 100 ns is shown in Figure 2. The maximum values in the curve correspond to the CFs. Both Equations (10) and (15) show that the CF is a key indicator because it is directly related to the TOF difference of the two separated shear waves. Hence, the CF can be collected by determining the maximum value in the curve of the DPD function.

### 2.4. Uniaxial Stress Measurement Using the Shear-Wave Pulse Echo Spectrum

According to the acoustoelastic effect, the velocities of the shear waves are different when their particle vibration directions are perpendicular and parallel to the stress direction, respectively. The velocities of the shear waves can be related to the uniaxial stress, which theoretical formulas can be found in references [24,50]. By further combining Equations (10) and (15), we obtain the following formula [24].

(18) $$\sigma =\frac{\kappa }{f^{*}}-\gamma ,\;(N_{2}=1),$$

(19) $$\kappa =\frac{2N_{1}-1}{2t_{0}}\cdot \frac{-8\mu^{2}}{4\mu +n},\;(N_{1}=N_{3}=1,\;2,\;3,\;...),$$

(20) $$\gamma =\frac{-8\mu^{2}}{4\mu +n}\alpha ,$$

where *σ* is the uniaxial stress in the direction of *x*_2_; *λ* and *μ* are the second-order elastic constants; *l*, *m*, and *n* are the third-order elastic constants; *t*_0_ is the shear-wave TOF in the free-stressed state and equals 2*l*/*v*_0_; *α* is a factor to indicate the initial anisotropy of the materials; and *κ* and *γ* should be fitted using the uniaxial compressive test.

## 3. Experimental Studies

### 3.1. Equipment and Sample

**Figure 3:**
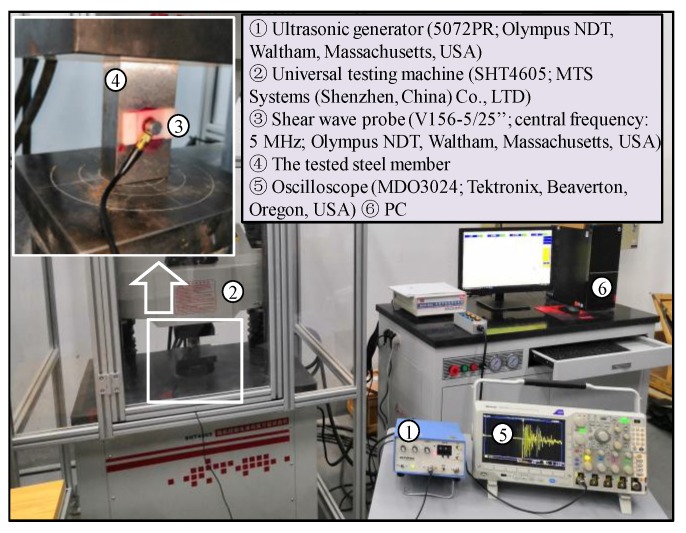
Measurement devices: photographs.

**Figure 4:**
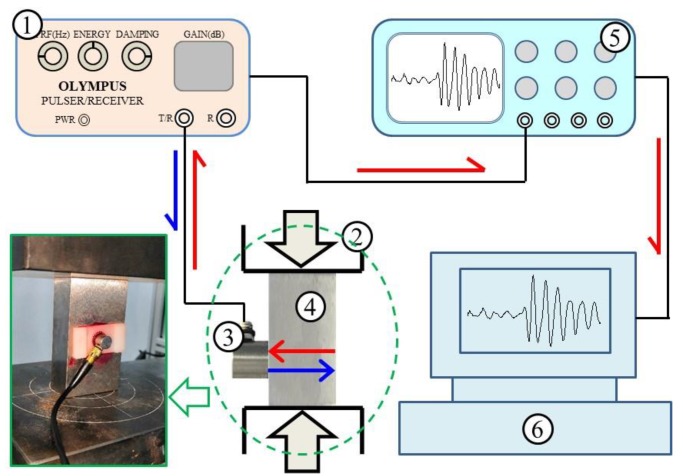
Measurement system: schematic diagram.

The devices and the measurement schematic diagram for identifying the uniaxial stress effect on the spectrum of the shear wave are shown in Figure 3 and Figure 4, respectively. The probe used in the experiments is a normal incidence shear-wave transducer (V156-5/25’’; central frequency: 5 MHz; Olympus NDT, Waltham, MA, USA), which can introduce shear waves directly into the steel member without the use of refraction. The shear-wave transceiver probe is excited by the ultrasonic generator (5072PR; Olympus NDT, Waltham, MA, USA) and a pure shear wave is generated. The generated shear wave is perpendicularly incident on the steel member loaded by a universal testing machine (SHT4605; MTS Systems (Shenzhen, China) Co., LTD). After being reflected from the rear side of the steel member, the shear wave travels back to the steel member surface and is received by the transceiver probe. The shear-wave pulse echo signals travel back to the ultrasonic generator and are finally collected by the oscilloscope (MDO3024; Tektronix, Beaverton, OR, USA). A personal computer (PC) is used to process the received signals. The polarized angle of the shear wave can be determined by rotating the transceiver probe that is imbedded in a card slot. More details of collecting the pulse echo shear waves can be found in paper [24].

Three steel members, made of Q235 steel, are designed and used as the test specimens. The dimensions of the three steel members are 80 mm × 45 mm × 24 mm (sample C1), 80 mm × 45 mm × 30 mm (sample C2), and 80 mm × 45 mm × 36 mm (sample C3). GW-type-III ultrasound coupler is used as the couplant to couple the probe and the specimens. 

As the shear-wave length is small enough (approximately 0.64 mm) and the shear-wave travel length is short enough (24 mm to 36 mm), we do not consider the influence of steel member boundaries on shear-wave propagation. Therefore, guided waves are not formed when the shear wave propagates in steel members. The experiment was conducted at room temperature (25 °C), and the temperature was considered constant. Therefore, variations in the operational temperature were not considered.

### 3.2. Influence of the Uniaxial Stress on the Shear-Wave Amplitude Spectrum

**Figure 5:**
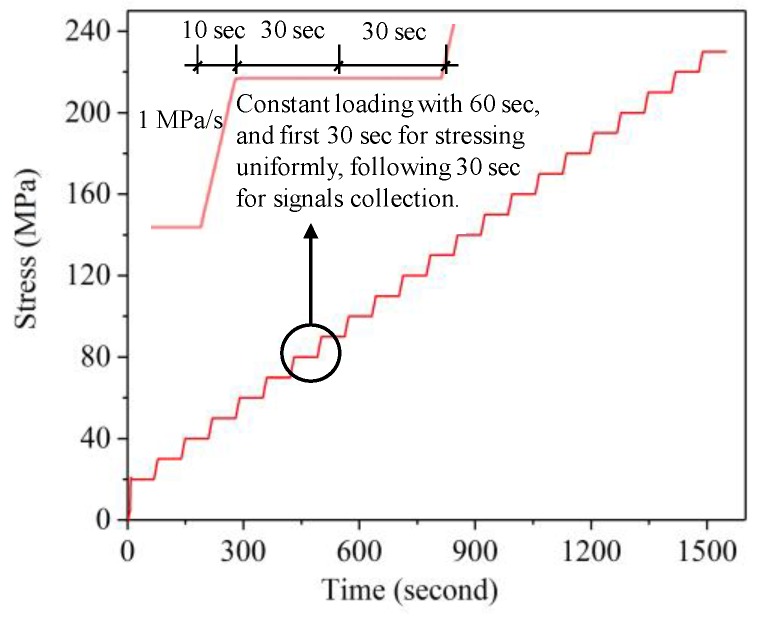
The increasing step load history applied on the three specimens.

**Figure 6:**
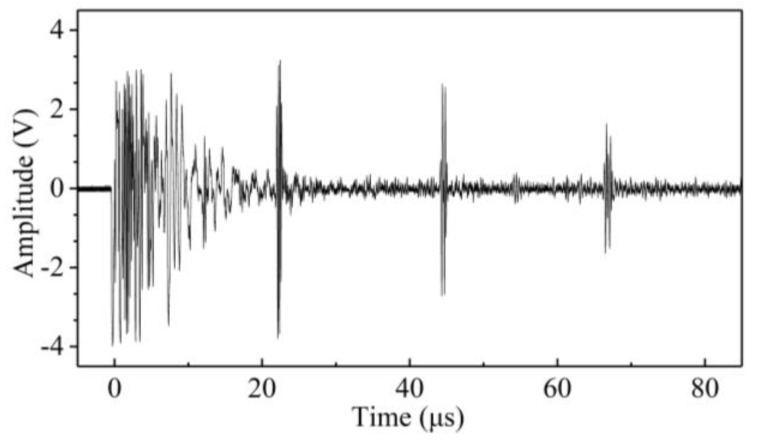
The typical time-domain signals of the received shear waves (sample C3, *σ* = 200 MPa).

A universal testing machine was used as the loading device to apply compressive stress along the vertical axis of the three specimens. The increasing step load history applied to the three specimens is shown in Figure 5. The step load increased from 20 MPa to 230 MPa with a step amplitude of 10 MPa. The transceiver probe is attached to the specimens’ surface. During each loading stabilization, the shear-wave pulse echo signals are collected using the oscilloscope with a sampling rate of 100 MSa/s. The typical time-domain signals of the received shear waves (sample C3, σ = 200 MPa) are shown in Figure 6. Using the Fourier transform method, the pulse echo signal can be converted into frequency domain signals [24].

**Figure 7:**
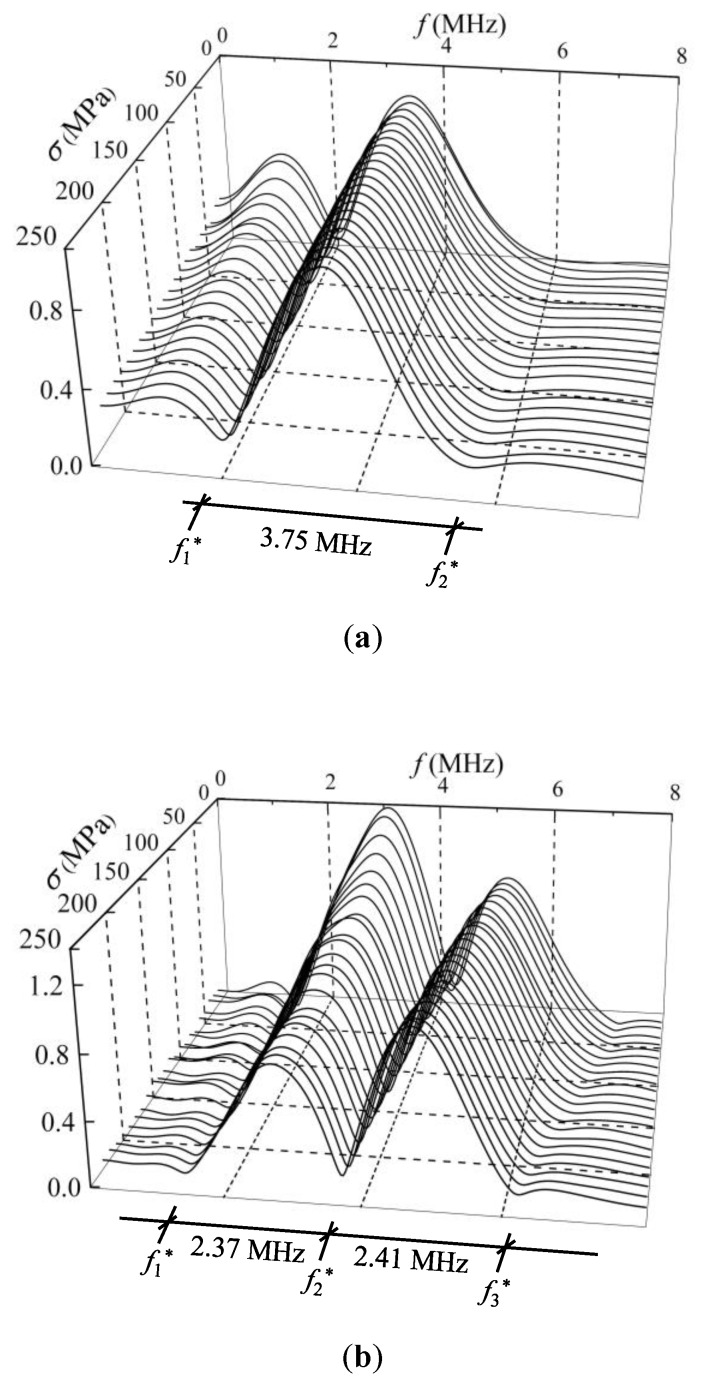

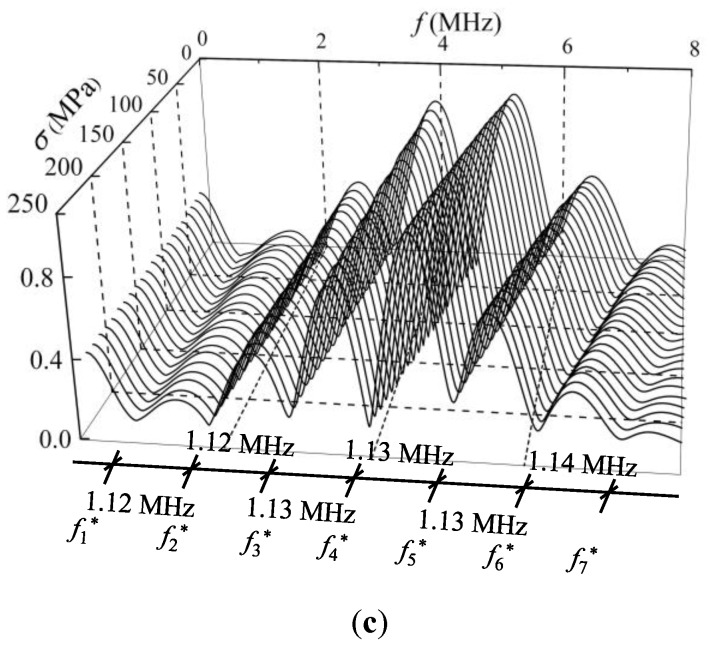
The normalized amplitude spectra under different compressive stress states in (**a**) sample C1; (**b**) sample C2; and (**c**) sample C3.

The shear wave is perpendicularly incident on the steel member with a polarized angle of 45°. The second pulse echo signals are extracted, and the corresponding applied uniaxial stresses are recorded. The Fourier transform method is used to transform the time domain signal to the amplitude spectrum. Hence, the change in the amplitude spectra affected by the stresses for the three specimens can be obtained. Figure 7 shows the normalized amplitude spectra under different compressive stress states in samples C1, C2, and C3.

### 3.3. Influence of Uniaxial Stress on the Shear-Wave Phase Spectrum

**Figure 8:**
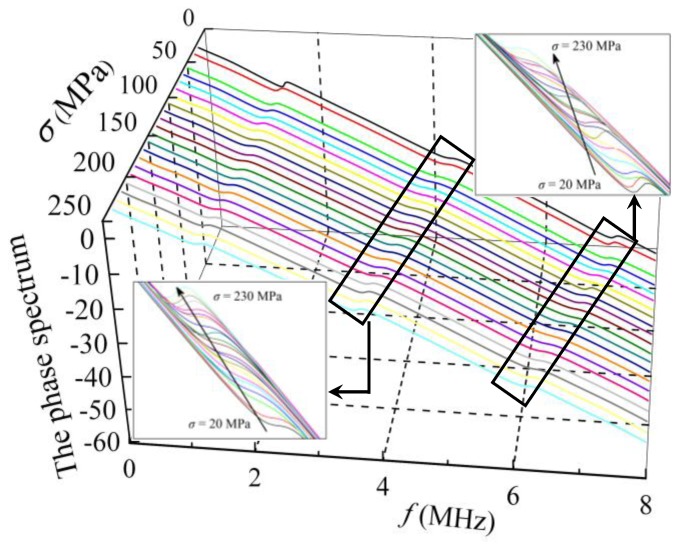
The change in the phase difference curves affected by the stress (*θ* = 40°).

The polarized angles of 40° and 45° were selected during the experiments. Using the Fourier transform method, the shear-wave phase spectrum could be obtained from the collected time-domain signals. The phase spectrum corresponding to different uniaxial compressive stresses could be obtained for the three specimens. A typical illustration of the change in the phase spectrum affected by the stress state is shown in Figure 8.

**Figure 9:**
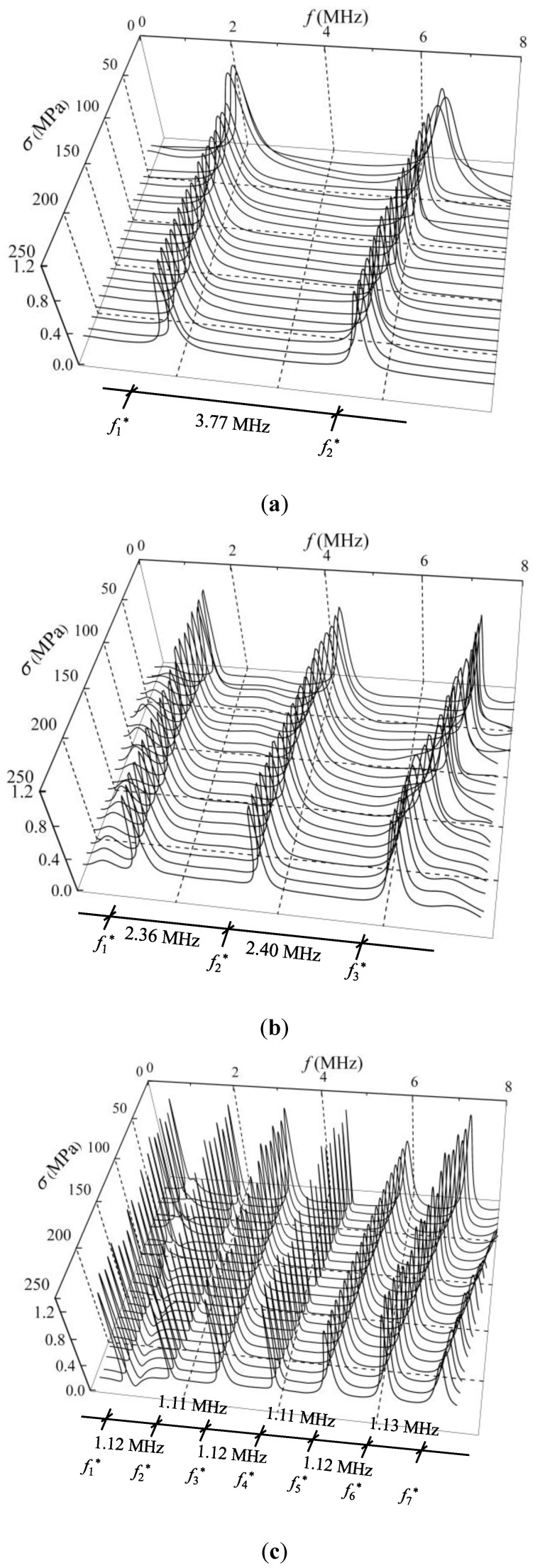
The normalized derivation of the phase difference curves under different compressive stress states in (**a**) sample C1; (**b**) sample C2; and (**c**) sample C3.

The PD describes the difference of the two phase spectra for an arbitrary polarized angle and a polarized angle of 45°. With the phase spectra of polarized angles at 40° and 45°, the PD under different stress states could be sketched. Further, the DPD curves was successfully obtained. The influence of stress on the normalized DPD curves for the three specimens is shown in Figure 9.

### 3.4. Parameter Calibration of the Stress Measurement Formula

**Figure 10:**
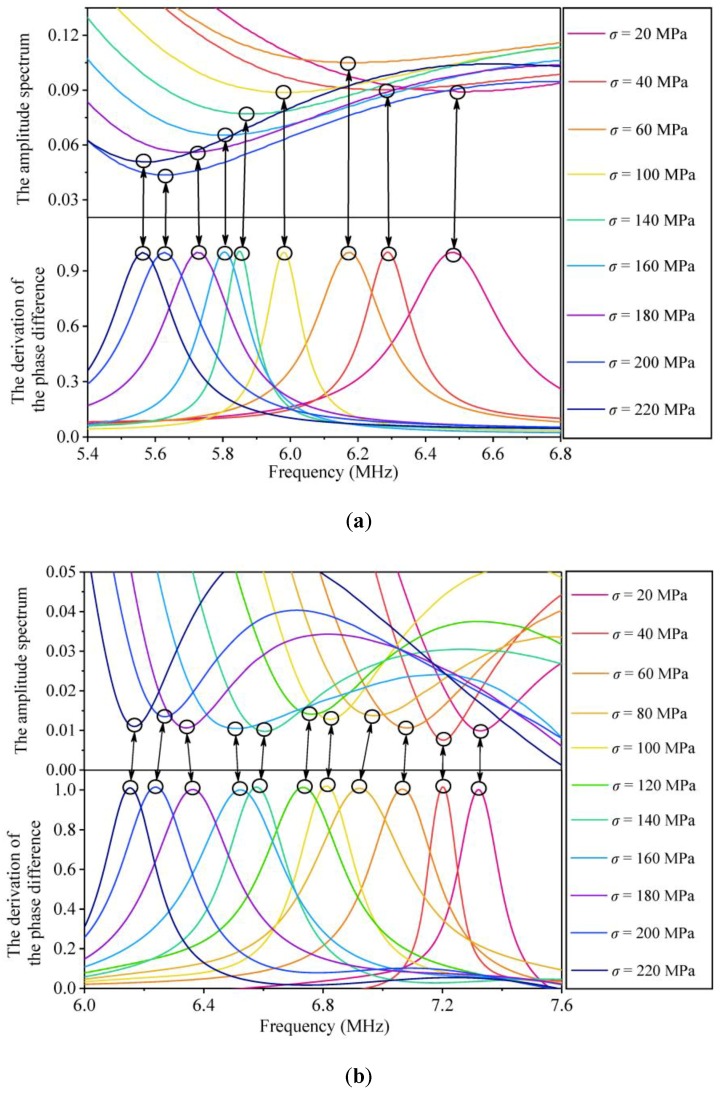

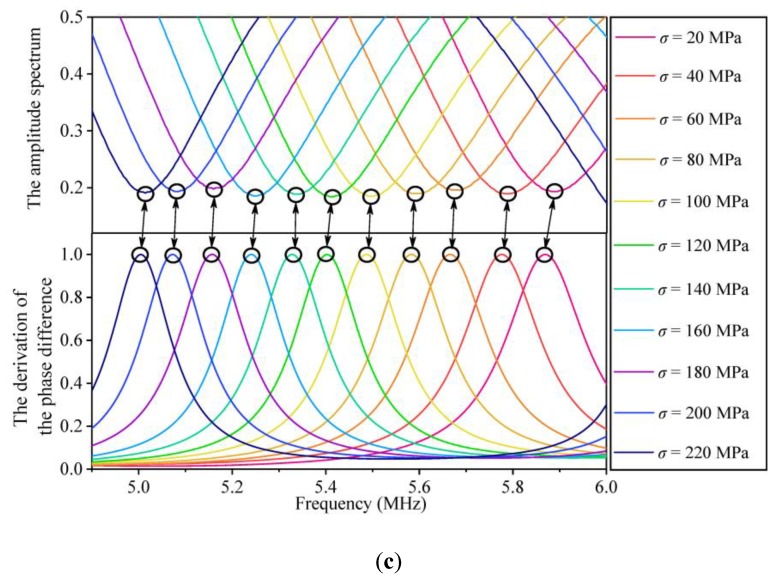
Comparison of the amplitude spectra and the derivation of the phase difference curves: (**a**) sample C1; (**b**) sample C2; and (**c**) sample C3.

**Figure 11:**
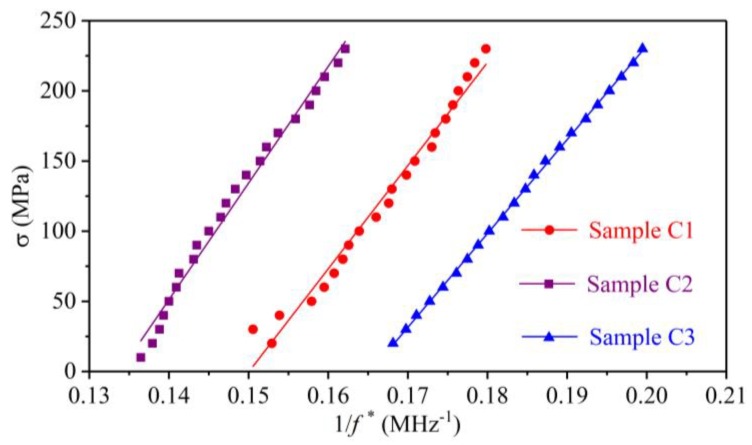
Fitting lines between the stress and the inverse of the characteristic frequency extracted from the amplitude spectra.

**Figure 12:**
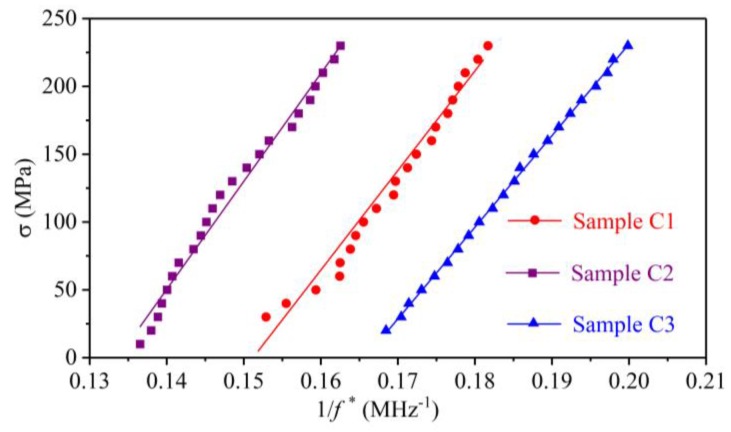
Fitting lines between the stress and the inverse of the characteristic frequency extracted from the derivation of the phase difference curves.

The aim of sketching the amplitude spectra and the DPD curves is to collect the CFs in each stress state. For the convenience of making a comparison between the amplitude spectra and the DPD curves, the second CFs for sample C1, the third CFs for sample C2, and the fifth CFs for sample C3 are extracted from the amplitude spectra and DPD curves under identical applied uniaxial stresses. The comparison of the CFs in the amplitude spectra and the DPD curves for the three samples are shown in Figure 10. Using the least squares method listed in reference [24], the parameters in Equation (18) can be obtained from a linear fitting of the stress and the inverse of the CF, which is shown in Figure 11 and Figure 12. The coefficients of the fitting line are listed in Table 1.

## 4. Results and Discussion

### 4.1. Influence of Uniaxial Stress on Shear-Wave Amplitude Spectrum

The amplitude spectrum of a shear wave traveling in a steel member under different stress state is definitely different, as shown in Figure 7. The main reason is that the interference effect between the two separated shear waves lead to an energy loss of the harmonic components. In particular, the energy of the harmonic component corresponding to the CF decreased to 0 when the shear-wave polarized angle reached 45°. In addition, the amplitude spectra did not change with stress when the shear-wave polarized angle was 0°. The reason for this phenomenon is that the velocities of the two separated shear waves are identical. Therefore, no interference occurs between the two separated shear waves. Another explanation of this phenomenon can be found in Equations (8) and (9), in which the AIF equals 1 when the shear-wave polarized angle is 0°. Hence, the pulse echo amplitude spectrum does not change with stress.

As shown in Figure 7, the minimum point is periodically presented in the amplitude spectra with a repetition period of 3.75 MHz, 2.39 MHz, and 1.25 MHz for samples C1, C2, and C3, respectively. According to Equation (10), the periodic values for the CF are 1/*P*. Because the TOF difference (*P*) of the two separated shear waves depends on the dimensions of the sample, sample C3 with the thickest size shows the least periodic value, while sample C1 with the thinnest size presents the maximal period. The minimum point in an amplitude spectrum corresponds to the CF. The CF shows a tendency to move left with increasing stress, which lays the foundation for stress evaluation. This is consistent with the theoretical analysis result in Equation (10): the CF decreases when the stress-induced TOF difference *P* increases.

### 4.2. Influence of Uniaxial Stress on Shear-Wave Phase Spectrum

Figure 8 shows the typical phase spectra when the applied compressive stress increases from 20 MPa to 230 MPa. All the phase spectra have a tendency to decrease with increasing frequency. The inflection points periodically appear on the phase spectra curves. With increasing compressive stress, the inflection points tend to move left. However, it is difficult to observe any quantitative relationship between the inflection points and the stresses.

The DPD curves of the three samples in Figure 9 show an obvious variation tendency. Compared with the phase spectrum, the influence of stress on the DPD curves is obvious because a peak point appears, which corresponds to a specific inflection point. The peak points periodically appeared in each DPD curve with nearly identical periods to the amplitude spectra for the three samples. As the peak point in the DPD curve corresponds to the CF, the CF can be obtained from the DPD curve. In Figure 9, the DPD curves of three samples show that the CF decreases with stress, which is consistent with the results obtained from the amplitude spectrum.

### 4.3. Comparison of the Amplitude and Phase Spectra

Equations (10) and (15) indicate that the abscissas corresponding to the minimum point in the amplitude spectrum and the maximum point in the DPD curve should be identical, which was verified by the experimental results in Figure 10. The CFs obtained from the amplitude spectra and the DPD curves in Figure 10 are nearly identical. Therefore, both the amplitude spectrum and the phase spectrum can be used to extract the CF. The difference between the two methods is that the stress exerts a direct influence on the amplitude spectrum, while the effect of stress on the phase spectrum is difficult to observe, and the CF is extracted from the DPD curve.

For the amplitude spectrum, the CF can be obtained with only one shear-wave pulse echo signal with a polarized angle of 45°. For the phase spectrum, two shear-wave pulse echo signals are required: one is the signal with a polarized angle of 45°, and the other is the signal with a polarized angle of close to 45°. Therefore, the shear-wave pulse echo signal with a polarized angle of 45° is required for both the amplitude and phase spectra.

Notably, the essence of the stress effect on the two spectra is identical, that is, the interference effect of the two separated shear waves. The velocities of the two separated shear waves propagating in a stressed steel member are different; thus, the interference effect occurs for the two separated shear waves. For the amplitude spectra, when the shear-wave polarized angle is 45°, the interference effect induces the amplitude of the CF to decrease to 0; thus, a minimum point appears, and the CF can be obtained. For the phase spectra, the maximum point in the DPD curve corresponds to the CF, and it changes with stress. Although the method of collecting the CF is different, the value of the CF corresponding to a certain stress state is identical. Therefore, the calibrated parameters using the two sets of data should be identical.

Note that the CF corresponding to a peak point in the DPD curve is easily observed, while the minimum point in the amplitude spectrum is not always obvious, as shown in Figure 7 and Figure 9. For instance, the second CFs (*f*_2_^*^) for sample C1 and the third CFs (*f*_3_^*^) for sample C2, which are shown in Figure 7a,b, are difficult to collect. The main reason is that the amplitude spectrum indicates the energy amplitude of a certain frequency, which is directly related to the transceiver probe. In this work, the central frequency of the probe is approximately 5 MHz, and the amplitude spectrum energy is centered on the range of 3–7 MHz. When the CFs are beyond the range of 3–7 MHz, the change in the amplitude spectrum energy is not dramatic and is difficult to observe. Therefore, from the aspect of CF extraction, the phase spectrum method is more advantageous.

### 4.4. Parametric Calibration for the Stress Measurement Formula

Figure 11 and Figure 12 show that the inverse of the CFs obtained from the amplitude spectra and the DPD curves linearly change with the stress. The calibrated parameters in Table 1 indicate that the correlation coefficients (*R*^2^) of all the lines are larger than 0.95, which verifies the correctness of Equation (18).

The difference in the calibrated parameter *κ* using the amplitude spectrum and the phase spectrum is less than 1% for the three samples. This error may come from the ambient effect and can be ignored. The parameter *γ* is related to the material and the initial acoustic anisotropy. Because the three samples were cut from one steel plate, the calibrated parameter *γ* is nearly identical for the three samples using the two types of spectra, in which the maximum error is 13.75 MPa. With the calibrated parameters, the uniaxial stress in a steel member can be evaluated by collecting the shear-wave pulse echo signals and extracting the CFs from the amplitude spectra or the DPD curves. The results in this work provide a potential way to detect the uniaxial absolute stress in structural steel members using the amplitude spectrum method and the phase spectrum method.

The experiments of this work were implemented on the laboratory scale, and a perfect linear relationship between the stress and the inverse of CFs was obtained. However, some necessary factors need to be considered for the absolute stress evaluation of realistic structures. For instance, the ambient temperature of realistic structures is uncontrollable, the surface roughness of the tested steel members will be notable due to the development of corrosion, and the coupling state between the probe and steel member surface is difficult to maintain in a constant state. All of these factors may exert a direct influence on the absolute stress measurement. Another limitation of the two methods is that structural steel members are usually non-removable after installation. It is hard to calibrate the parameters on the original tested member. A possible solution is to replicate a steel member with the same material and dimensions as the tested steel member. However, the replicated steel member is not the original in-service steel member, and the potential differences between the tested and replicated steel members may lead to errors in parametric calibration. To realize the practical engineering application of the shear-wave spectrum method, further research efforts should focus on the influence of temperature, surface roughness and coupling state on parametric calibration and the absolute stress measurement.

## 5. Conclusions

In this paper, the influence of uniaxial stress on the shear-wave spectrum propagating in steel members was investigated. Three steel members were used to study the effect of the applied uniaxial stress on the amplitude spectrum and phase spectrum. The conclusions are summarized as follows:

(1) The theoretical expressions of the shear-wave pulse echo phase spectrum were derived. The essence of the stress effect on the shear-wave phase spectrum is the interference effect of the two separated shear waves, which follows the same principles as the shear-wave amplitude spectrum.

(2) The CFs can be obtained from both the amplitude spectrum and the DPD curve. The extracted CFs are identical when the steel member is under the same stress state. To collect a CF, one shear-wave signal is required for the amplitude spectrum, while two shear-wave signals are needed for the phase spectrum.

(3) The inverse of the CF showed a linear relationship with the corresponding uniaxial stress, thus establishing a basis for the uniaxial stress evaluation. The calibrated parameters obtained from the two methods are nearly identical. Using the calibrated parameters, the uniaxial stress in a steel member can be evaluated by extracting the CF from the shear-wave pulse echo signal.

## Figures and Tables

**Table 1:** Coefficients of the fitting line using the amplitude spectrum and the phase spectrum.

Parameters	Methods	Sample C1		Sample C2		Sample C3	
		Values	Difference	Values	Difference	Values	Difference
*κ* (MPa·MHz)	Amplitude Spectrum	7356.73	0.23%	8308.07	0.16%	6691.01	0.01%
	Phase Spectrum	7339.74		8321.41		6755.27	
*γ* (MPa)	Amplitude Spectrum	1103.99	5.83	1111.95	6.06	1106.05	13.75
	Phase Spectrum	1109.82		1118.01		1119.80	
*R* ^2^	Amplitude Spectrum	0.9901	/	0.9937	/	0.9998	/
	Phase Spectrum	0.9795	/	0.9828	/	0.9996	/

## References

[1] Nichols J.M. (2003). Structural health monitoring of offshore structures using ambient excitation. Appl. Ocean Res..

[2] Li J., Hao H., Fan K., Brownjohn J. (2015). Development and application of a relative displacement sensor for structural health monitoring of composite bridges. Struct. Control Health Monit..

[3] Biondini F., Frangopol D.M. (2016). Life-cycle performance of deteriorating structural systems under uncertainty: Review. J. Struct. Eng..

[4] Wang H., Li G., Huang X. (2016). Behavior of coupled shear walls with buckling-restrained steel plates in high-rise buildings under lateral actions. Struct. Des. Tall Spec..

[5] Wang J., Zhao H. (2018). High performance damage-resistant seismic resistant structural systems for sustainable and resilient city: A review. Shock Vib..

[6] Sousa H., Wang Y. (2018). Sparse representation approach to data compression for strain-based traffic load monitoring: A comparative study. Measurement.

[7] Asadollahi P., Huang Y., Li J. (2018). Bayesian finite element model updating and assessment of cable-stayed bridges using wireless sensor data. Sensors.

[8] Luo M.Z., Li W.J., Wang B., Fu Q.Q., Song G.B. (2017). Measurement of the length of installed rock bolt based on stress wave reflection by using a giant magnetostrictive (GMS) actuator and a PZT sensor. Sensors.

[9] Laflamme S., Kollosche M., Connor J.J., Kofod G. (2012). Soft capacitive sensor for structural health monitoring of large-scale systems. Struct. Control Health Monit..

[10] Yi T.H., Li H.N., Gu M. (2013). Recent research and applications of GPS-based monitoring technology for high-rise structures. Struct. Control Health Monit..

[11] Ay A.M., Wang Y. (2014). Structural damage identification based on self-fitting ARMAX model and multi-sensor data fusion. Struct. Health Monit..

[12] Teng J., Lu W., Wen R.F., Zhang T. (2015). Instrumentation on structural health monitoring systems to real world structures. Smart Struct. Syst..

[13] Meoni A., D’Alessandro A., Downey A., García-Macías E., Rallini M., Materazzi A.L., Torre L., Laflamme S., Castro-Triguero R., Ubertini F. (2018). An experimental study on static and dynamic strain sensitivity of embeddable smart concrete sensors doped with carbon nanotubes for SHM of large structures. Sensors.

[14] Shen Y.B., Yang P.C., Zhang P.F., Luo Y.Z., Mei Y.J., Cheng H.Q., Jin L., Liang C.Y., Wang Q.Q., Zhong Z.N. (2013). Development of a multitype wireless sensor network for the large-scale structure of the National Stadium in China. Int. J. Distrib. Sens. N..

[15] Li G.W., Pei H.F., Yin J.H., Lu X.C., Teng J. (2014). Monitoring and analysis of PHC pipe piles under hydraulic jacking using FBG sensing technology. Measurement.

[16] Teng J., Lu W., Cui Y., Zhang R.G. (2016). Temperature and displacement monitoring to steel roof construction of Shenzhen Bay Stadium. Int. J. Struct. Stab. Dyn..

[17] Suzuki K. (2017). Proposal for a direct-method for stress measurement using an X-ray area detector. NDT E Int..

[18] Hemmesi K., Farajian M., Boin M. (2017). Numerical studies of welding residual stresses in tubular joints and experimental validations by means of x-ray and neutron diffraction analysis. Mater. Des..

[19] Zhou D., Pan M., He Y., Du B. (2017). Stress detection and measurement in ferromagnetic metals using pulse electromagnetic method with U-shaped sensor. Measurement.

[20] Rossini N.S., Dassisti M., Benyounis K.Y., Olabi A.G. (2012). Methods of measuring residual stresses in components. Mater. Des..

[21] Liu T.J., Zou D.J., Du C.C., Wang Y. (2017). Influence of axial loads on the health monitoring of concrete structures using embedded piezoelectric transducers. Struct. Health Monit..

[22] Li Z.H., He J.B., Teng J., Wang Y. (2016). Internal stress monitoring of in-service structural steel members with ultrasonic method. Materials.

[23] He J.B., Li Z.H., Teng J., Wang Y. (2018). Absolute stress field measurement in structural steel members using the Lcr wave method. Measurement.

[24] Li Z.H., He J.B., Teng J., Huang Q., Wang Y. (2017). Absolute stress measurement of structural steel members with ultrasonic shear-wave spectral analysis method. Struct. Health. Monit..

[25] Li Z.H., He J.B., Teng J., Wang Y. (2018). Cross-correlation-based algorithm for absolute stress evaluation in steel members using the longitudinal critically refracted wave. Int. J. Distrib. Sens. Netw..

[26] Withers P.J., Turski M., Edwards L., Bouchard P.J., Buttle D.J. (2008). Recent advances in residual stress measurement. Int. J. Press. Vessel. Pip..

[27] Guz’ A.N., Makhort F.G. (2000). The physical fundamentals of the ultrasonic nondestructive stress analysis of solids. Int. J. Appl. Mech..

[28] Bray D.E., Tang W. (2001). Subsurface stress evaluation in steel plates and bars using the L-CR ultrasonic wave. Nucl. Eng. Des..

[29] Karabutov A., Devichensky A., Ivochkin A., Lyamshevb M., Pelivanova I., Rohadgic U., Solomatina V., Subudhic M. (2008). Laser ultrasonic diagnostics of residual stress. Ultrasonics.

[30] Egle D.M., Bray D.E. (1976). Measurement of acoustoelastic and 3rd-order elastic-constants for rail steel. J. Acoust. Soc. Am..

[31] Lee H.Y., Nikbin K.M., O’Dowd N.P. (2005). A generic approach for a linear elastic fracture mechanics analysis of components containing residual stress. Int. J. Press. Vessel. Pip..

[32] Sanderson R.M., Shen Y.C. (2010). Measurement of residual stress using laser-generated ultrasound. Int. J. Press. Vessel. Pip..

[33] Javadi Y., Najafabadi M.A. (2013). Comparison between contact and immersion ultrasonic method to evaluate welding residual stresses of dissimilar joints. Mater. Des..

[34] Sadeghi S., Najafabadi M.A., Javadi Y., Mohammadisefat M. (2013). Using ultrasonic waves and finite element method to evaluate through-thickness residual stresses distribution in the friction stir welding of aluminum plates. Mater. Des..

[35] Chaki S., Bourse G. (2009). Stress level measurement in prestressed steel strands using acoustoelastic effect. Exp. Mech..

[36] Gandhi N., Michaels J.E., Lee S.J. (2012). Acoustoelastic Lamb wave propagation in biaxially stressed plates. J. Acoust. Soc. Am..

[37] Wali Y., Njeh A., Wieder T., Ghozlen M.B. (2007). The effect of depth-dependent residual stresses on the propagation of surface acoustic waves in thin Ag films on Si. NDT E Int..

[38] Wang Z.J., Chen D.D., Zheng L.Q., Huo L.S., Song G.B. (2018). Influence of axial load on electromechanical impedance (emi) of embedded piezoceramic transducers in steel fiber concrete. Sensors.

[39] Allen D.R., Sayers C.M. (1984). The measurement of residual-stress in textured steel using an ultrasonic velocity combinations technique. Ultrasonics.

[40] Lipeles R., Kivelson D. (1977). Theory of ultrasonically induced birefringence. J. Chem. Phys..

[41] Crecraft D.I. (1967). The measurement of applied and residual stresses in metals using ultrasonic waves. J. Sound Vib..

[42] Herzer H.R., Becker M.M., Schneider E., Ida N., Meyendorf N. (2018). The acousto-elastic effect and its use in NDE. Handbook of Advanced Non-Destructive Evaluation.

[43] Djerir W., Ourak M., Boutkedjirt T. (2014). Characterization of the critically refracted longitudinal (L-CR) waves and their use in defect detection. Res. Nondestruct. Eval..

[44] Palanichamy P., Joseph A., Jayakumar T., Raj B. (1995). Ultrasonic velocity measurements for estimation of grain size in austenitic stainless steel. NDT E Int..

[45] Lhémery A., Calmon P., Chatillon S., Gengembre N. (2002). Modeling of ultrasonic fields radiated by contact transducer in a component of irregular surface. Ultrasonics.

[46] Zou D.J., Liu T.J., Liang C.F., Huang Y.C., Zhang F.Y., Du C.C. (2015). An experimental investigation on the health monitoring of concrete structures using piezoelectric transducers at various environmental temperatures. J. Intell. Mater. Syst. Struct..

[47] Liu H.B., Li Y.P., Li T., Zhang X., Liu Y.K., Liu K., Wang Y.Q. (2018). Influence factors analysis and accuracy improvement for stress measurement using ultrasonic longitudinal critically refracted (LCR) wave. Appl. Acoust..

[48] Vangi D., Virga A. (2007). A practical application of ultrasonic thermal stress monitoring in continuous welded rails. Exp. Mech..

[49] Blinka J., Sachse W. (1976). Application of ultrasonic-pulse-spectroscopy measurements to experimental stress analysis. Exp. Mech..

[50] Javadi Y., Azari K., Ghalehbandi S.M., Roy M.J. (2017). Comparison between using longitudinal and shear waves in ultrasonic stress measurement to investigate the effect of post-weld heat-treatment on welding residual stresses. Res. Nondestruct. Eval..

